# Secrecy Performance Maximization for Underlay CR Networks with an Energy Harvesting Jammer

**DOI:** 10.3390/s21248198

**Published:** 2021-12-08

**Authors:** Juan Sun, Shubin Zhang, Kaikai Chi

**Affiliations:** School of Computer Science and Technology, Zhejiang University of Technology, Hangzhou 310023, China; 1111912009@zjut.edu.cn (J.S.); kkchi@zjut.edu.cn (K.C.)

**Keywords:** physical layer security, CR networks, RF energy-harvesting, DC programming, Lagrange duality method

## Abstract

This paper investigates the secrecy communication in an underlay cognitive radio (CR) networks with one primary user (PU) as well as multiple PUs, where the radio frequency (RF) energy-harvesting secondary user (SU) transmits the confidential information to the destination in the presence of a potential eavesdropper. We introduce a RF energy-harvesting secondary jammer (SJ) to secure the SU transmissions. The system works in time slots, where each time slot is divided into the energy transfer (ET) phase and the information transfer (IT) phase. In ET phase, the SU and SJ capture energy from the PU transmissions; in the IT phase, the SU uses the harvested energy to transmit information to the destination without causing the harmful interference to the PU transmissions, while the SJ utilizes the captured energy to generate jamming signals to the eavesdropper to secure the SU transmissions. We aim to maximize the secrecy rate for SU transmissionsby jointly optimizing the time allocation between ET phase and IT phase and the transmit power allocation at the SU and SJ. We first formulate the secrecy rate maximization as non-convex optimization problems. Then, we propose efficient nested form algorithms for the non-convex problems. In the outer layer, we obtain the optimal time allocation by the one dimension search method. In the inner layer, we obtain the optimal transmit power allocation by the DC programming, where the Lagrange duality method is employed to solve the convex approximation problem. Simulation results verify that the proposed schemes essentially improve the secrecy rate of the secondary network as compared to the benchmark schemes.

## 1. Introduction

### 1.1. CR Networks with RF Energy-Harvesting

To fulfill ever-increasing demands for wireless services and applications, cognitive radio (CR) technology has been emerged to lighten severe shortage of spectrum resources [1]. CR technology allows the secondary users (SUs) to access the spectrum licensed to the primary users (PUs), based on the premise that the quality of service (QoS) requirement of the PUs must be guaranteed [2,3]. In the CR networks, underlay and overlay are two main schemes that the SUs share the spectrum with the PUs [4,5]. In the underlay paradigm, the SUs transmit concurrently in the same spectrum with the PUs, if the interference caused by the SUs to the PUs is below a given threshold. On the other hand, the explosion of wireless services and applications has also led to energy deficiency in the future wireless communications. Radio-frequency (RF) energy-harvesting technology is a good candidate solution for charging the low-power wireless devices [6,7,8,9,10], which can conquer the uncontrollability and intermittency of wireless devices powered by the renewable energy sources, such as wind, solar and vibrational energy. The harvest-then-transmit (HTT) protocol is proposed in [11] as one of the most common working mode of RF energy-harvesting technology, where the users first harvest energy from the RF energy source in the energy transfer (ET) phase and then use the captured energy to transmit information to the destination in the information transfer (IT) phase.

In order to simultaneously improve the energy efficiency and spectrum efficiency mentioned above, the use of RF energy-harvesting technology in CR networks has been studied extensively in the literature [12,13,14,15,16,17]. In [12], the authors presented an overview of RF energy-harvesting CR networks, where the SUs can capture energy from the PUs transmissions and transmit information when the channel is vacated by the PUs. The authors in [13] investigated the end-to-end throughput maximization problem by jointly optimizing the power and time allocation for the underlay CR networks. An underlay multi-hop CR networks with RF energy-harvest SUs was also considered in [14], where the authors derived the expressions of throughput and end-to-end outage probability. In [15], the authors studied optimal sensing intervals for an overlay RF energy-harvesting CR networks, where the unslotted PUs was considered. The authors in [16] found the optimal sensing policy to maximize the SU throughput subject to the energy constraint of the SU. In [17], the authors integrated ambient backscatter communication into RF energy-harvesting CR networks, where the analytic expressions for the average energy consumption, average achievable throughput and energy efficiency were derived.

### 1.2. Achieving Physical Layer Security of Wireless Networks

Besides the spectrum efficiency and energy efficiency, wireless communications are also facing the challenge of being wiretapped by the malicious eavesdroppers, due to the intrinsic broadcast nature of wireless medium. Physical layer security (PLS) is a promising solution to achieve the secure communication by exploiting physical properties of wireless channels, e.g., noise, fading, interference, etc [18]. The general goal of PLS is to maximize the secrecy rate, which is defined as the capacity of the main channel minus that of the wiretap channel.

To enhance physical layer security, various techniques have been investigated in past decades, such as multiple-antenna technology, relay, and cooperation [19,20,21,22,23,24,25], etc. Cooperative jamming (CJ) as a practical approach among them has drawn much attention recently, which uses friendly jammer to generate jamming signals to impair the illegal link. The CJ strategy was proposed in [23] to deal with eavesdroppers anywhere in the wireless networks, where the jammer placement algorithms were analyzed. The authors in [24] maximized the secrecy rate at the destination under the transmit power constraints for the amplify-and-forward (AF) relay and the harvest-and-jam (HJ) helpers, where the multi-antenna HJ were used in a multi-antenna AF relay wiretap channel. Motivated by the RF energy-harvesting technology, the authors in [25] considered wireless powered jammer to secure the downlink communication from the source to the destination, where the jammer first harvested energy from the source and then generated jamming signals to the potential eavesdropper. The secrecy rate was maximized by jointly optimizing the time and power allocation. The wireless powered jammer was also introduced in [26], where the authors considered the secrecy rate and secrecy outage probability according to the level of channel state information (CSI) of the eavesdroppers, respectively.

### 1.3. Achieving PLS of CR Networks

In addition, CR networks are also vulnerable to malicious eavesdropping, which have drawn significant attention. The authors in [27] studied the spectrum access method and the PLS of CR networks, where some mathematical expressions for PLS performance metrics were derived. The authors in [28] proposed a game theory to optimize the secondary secrecy rate and the primary secrecy rate in CR networks, where the SU transmitted jamming signals for both the primary transmission and secondary transmission. In [29], the primary transmission privacy was guaranteed by introducing a wireless powered secondary jammer. Contrary to [29], the secondary transmission secrecy against eavesdropping by PUs was considered in [30]. Different from the conventional single-carrier CR networks, the authors in [31] considered secrecy energy efficiency (SEE) problem for a orthogonal frequency division multiplexing (OFDM)-based CR networks, where the instantaneous channel state information (ICSI) of the eavesdropper and the statistical CSI (SCSI) of the eavesdropper were both considered.

To date, few works have been done on PLS of RF energy-harvesting CR networks. Inspired by this, we investigate the PLS of underlay CR networks, where the SU and secondary jammer (SJ) harvest energy from the PU transmissions. The differences between our work and the previous woks [28,29] are summarized as follows: (a) The authors in [28,29] consider overlay CR networks with one PU, while we focus on underlay CR networks with one PU as well as multiple PUs. (b) In [28,29], the SU transmits artificial noise (AN) to confuse the eavesdropper, while a wireless powered cooperative jammer is introduced in our work to impair the wiretap channel. (c) The authors in [29] consider the secure transmissions for the primary network and the authors in [28] investigate secure transmissions for both the primary and secondary networks, while we study the secure transmission for the secondary networks. The main contributions of this paper are summarized as follows.

- We propose communication schemes for the underlay CR networks with one PU as well as multiple PUs. The SU and SJ first capture energy from the PU transmissions. Then, the SU transmits confidential information to the secondary receiver (SR), and the SJ simultaneously transmits jamming signals to confuse the eavesdropper. We study the secrecy rate maximization problem for the secondary network by jointly optimizing the time allocation between ET phase and IT phase, and the transmit power allocation for SU and SJ. We formulate the original problems as optimization problems which are non-convex.
- We design efficient nested form algorithms for both non-convex problems. In the outer layer, the time allocation is obtained by the one-dimension search approach. In the inner layer, the transmit power allocation is obtained by the DC programming, where the Lagrange duality method is employed to solve the convex approximation problem.
- Simulation results are given to verify the superiority of the proposed schemes as compared to the benchmark schemes. Moreover, we observe the impact of system parameter settings on secrecy performance.

## 2. System Model and Problem Formulation for Underlay CR Networks with One PU

### 2.1. System Model

**Figure 1:**
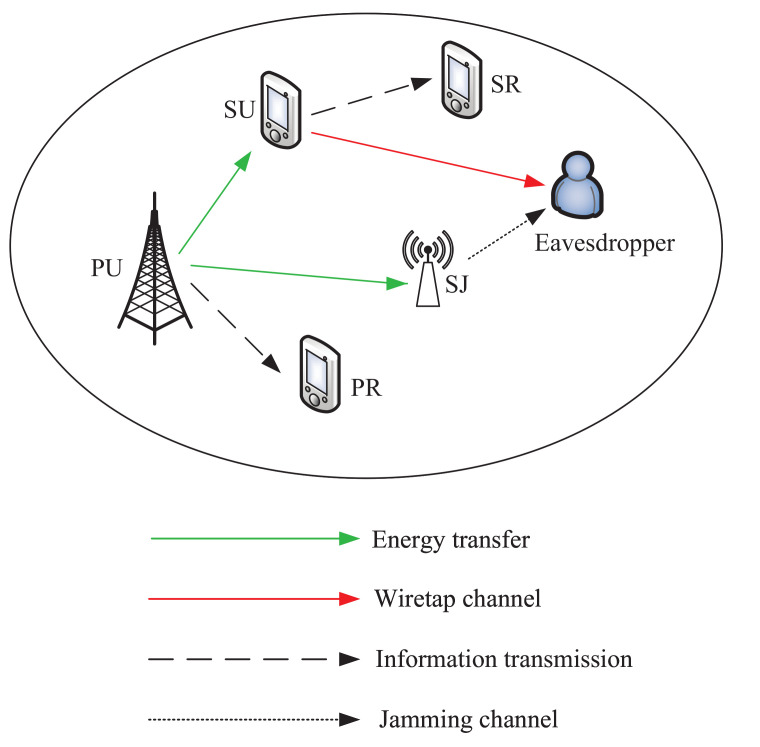
System model. The underlay CR networks with one PU, where SU uses the harvested energy to transmit confidential information to SR and SJ simultaneously employs the captured energy to confuse the eavesdropper.

The considered underlay CR networks is shown in Figure 1. The primary network consists of a PU licensed with spectrum, and a corresponding primary receiver (PR). In the secondary network, SU transmits information to a secondary receiver (SR) by accessing the PU spectrum, if it does not cause the harmful interference to the PU transmissions, where the confidential information is threated by a potential eavesdropper. We employ a SJ to secure the secondary network. SU and SJ have no embedded power supply, which need to harvest energy from the PU transmissions in the ET phase. We consider a block-based quasi-static channel model where the CSI remains constant over each time block, but changes from one time block to another. Specially, the CSI can be obtained via efficient channel estimation and feedback methods. Without loss of generality, we assume that the time block T is 1 s.

**Figure 2:**
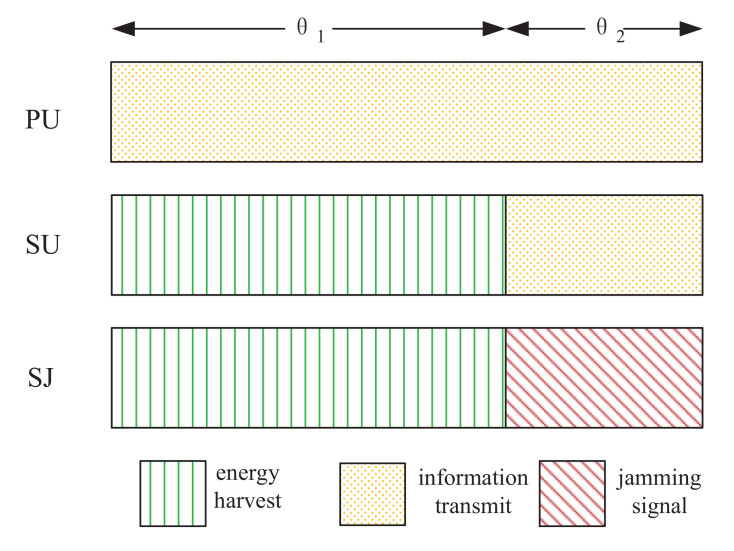
Block structure diagram. $\theta_{1}$ denotes the ET phase, during which SU and SJ harvest energy from the PU transmissions. $\theta_{2}$ denotes the IT phase, during which SU transmits confidential information to SR, and SJ generates jamming signals to the eavesdropper.

The specific block structure diagram can be referred to Figure 2. In the entire time block, PU transmits information to PR with a fixed power $P_{S}$. Furthermore, the entire time block is divided into two phases with lengths $\theta_{1}$ and $\theta_{2}$, which denote the ET phase and IT phase, respectively. Therefore, the time constraint should satisfy

(1) $$\theta_{1}+\theta_{2}\leq 1,\;\;0<\theta_{1}\leq 1,\;\;0\leq \theta_{2}<1.$$

Specially, the SJ keeps synchronously with the SU. That is, for SU and SJ, ET phase and IT phase must be consistent. In the EH phase, SU and SJ harvest energy from the PU transmissions. The energy harvested by the SU is expressed as

(2) $$E_{SU}=\theta_{1}\eta P_{S}h_{ps},$$

where η and $h_{ps}$ denote the energy harvesting efficiency at the SU and the channel power gain from the PU to SU, respectively. Similarly, the energy captured by SJ is expressed as

(3) $$E_{SJ}=\theta_{1}\eta P_{S}h_{pj},$$

where the energy harvesting efficiency at SJ is assumed to be the same as the SU [25,26], and $h_{pj}$ denotes the channel power gain from the PU to SJ.

In the IT phase, the SU uses the harvested energy $E_{SU}$ to transmit privacy information to SR. The total energy consumed by the SU to transmit information should satisfy

(4) $$\theta_{2}P_{SU}\leq \theta_{1}\eta P_{S}h_{ps},$$

where $P_{SU}$ denotes the transmit power at the SU. To guarantee the QoS requirement of the PU, the transmit power at the SU should be below a given threshold *I*, which is given by

(5) $$P_{SU}\leq I.$$

Meanwhile, the SJ simultaneously utilizes the captured energy $E_{SJ}$ to generate the jamming signals to the eavesdropper against its eavesdropping. The total energy consumed by the SJ to generate jamming signals should satisfy

(6) $$\theta_{2}P_{SJ}\leq \theta_{1}\eta P_{S}h_{pj},$$

where $P_{SJ}$ denotes the transmit power at the SJ. To guarantee the QoS requirement of PU, $P_{SJ}$ should also be below a given threshold *I*, which is expressed as

(7) $$P_{SJ}\leq I.$$

Based on the above analysis, the throughput $R_{SR}$ at SR and the throughput $R_{SE}$ at the eavesdropper, are expressed as

(8) $$R_{SR}=\theta_{2}W\log_{2}\left(1+\frac{P_{SU}h_{sr}}{\sigma_{S}^{2}}\right)$$

and

(9) $$R_{SE}=\theta_{2}W\log_{2}\left(1+\frac{P_{SU}h_{se}}{P_{SJ}h_{je}+\sigma_{E}^{2}}\right),$$

where *W* is the bandwidth, $\sigma_{S}^{2}$ and $\sigma_{E}^{2}$ denote the noise power at the SR and the eavesdropper, $h_{sr}$, $h_{se}$ and $h_{je}$ denote the channel power gains from SU to SR, SU to the eavesdropper, and SJ to the eavesdropper. Here, the CSI of the eavesdropper can be obtained by monitoring the possible transmissions of the eavesdropper as is commonly used in the PLS literature [25]. We assume that the jamming signals from the SJ is known at the SR, which means that the SJ interference can be removed at the SR [25,26].

Therefore, the secrecy rate of the secondary network is given by

(10) $$R_{sec}={\left[R_{SR}-R_{SE}\right]}^{+},$$

where ${\left[x\right]}^{+}=\max\left(x,0\right)$.

### 2.2. Problem Formulation

We target to maximize the secrecy rate in (10), subject to the time constraint in (1), the transmit power constraints in (5) and (7), and the energy causality constraints in (4) and (6). The decision variables include the time allocation $\theta_{1}$ and $\theta_{2}$, the transmit power allocation $P_{SU}$ at the SU, and the transmit power allocation $P_{SJ}$ at the SJ. Then, the secrecy rate maximization problem is formulated as

(11) (P1):maxθ1,θ2PSU,PSJθ2W[log2(1+PSUhsrσS2)−log2(1+PSUhsePSJhje+σE2)],s.t. (1), (4), (5), (6), (7).

Note that problem (P1) is non-convex as its objective function is non-concave. As a result, it is difficult to tackle in general.

## 3. Secrecy Rate Optimization Design for Underlay CR Networks with One PU

Firstly, we give Lemma 1 to reveal the property of the optimal time allocation solution.

**Lemma** **1.** *The optimal time allocation solution of problem (P1) uses up the entire time block, i.e., $\theta_{1}^{*}+\theta_{2}^{*}=1.$*

**Proof.**  Assume that the optimal time allocations of $\theta_{1}^{*}$ and $\theta_{2}^{*}$ satisfy $\theta_{1}^{*}+\theta_{2}^{*}<1$. Let us construct another new solution {$P_{SU}$, $P_{SJ}$, $\theta_{1}^{{}^{\prime }}$, $\theta_{2}^{{}^{\prime }}$}, with $\theta_{1}^{{}^{\prime }}=\beta \theta_{1}^{*}$ and $\theta_{2}^{{}^{\prime }}=\beta \theta_{2}^{*}$, where $\beta =\frac{1}{\theta_{1}^{*}+\theta_{2}^{*}}>1$. So $\theta_{1}^{{}^{\prime }}+\theta_{2}^{{}^{\prime }}=1$. Note that {$P_{SJ}$, $P_{SU}$, $\theta_{1}^{{}^{\prime }}$, $\theta_{2}^{{}^{\prime }}$} is also a feasible solution of problem (P1), which satisfies (1), (4), (5), (6) and (7). From (8), (9) and (10), we can see that when the IT phase $\theta_{2}^{{}^{\prime }}$ is larger, the secrecy rate $R_{sec}\left(\theta_{2}^{{}^{\prime }}\right)>R_{sec}\left(\theta_{2}^{*}\right)$, which contradicts to the assumption. Thus, we complete the proof.    □

Considering the non-convex of problem (P1), we solve it by formulating it into a nested form as

(12) $$\begin{array}{c} (P2):\;\max_{\theta_{2}}\;\;\theta_{2}WR\left(\theta_{2}\right),\\ s.t.\;\;\;0\leq \theta_{2}<1, \end{array}$$

where $R(\theta_{2})$ is expressed as

(13a) $$\begin{array}{cc} R\left(\theta_{2}\right)=\max_{P_{SU},P_{SJ}}\; & \log_{2}\left(1+\frac{P_{SU}h_{sr}}{\sigma_{S}^{2}}\right)-\log_{2}\left(1+\frac{P_{SU}h_{se}}{P_{SJ}h_{je}+\sigma_{E}^{2}}\right), \end{array}$$

(13b) $$\begin{array}{cc} s.t.\; & \theta_{2}P_{SU}\leq \left(1-\theta_{2}\right)\eta P_{S}h_{ps}, \end{array}$$

(13c) $$\begin{array}{cc} \; & P_{SU}\leq I, \end{array}$$

(13d) $$\begin{array}{cc} \; & \theta_{2}P_{SJ}\leq \left(1-\theta_{2}\right)\eta P_{S}h_{pj}, \end{array}$$

(13e) $$\begin{array}{cc} \; & P_{SJ}\leq I. \end{array}$$

Here, for the outer layer (12), the optimal time allocation $\theta_{2}$ can be obtained through one-dimensional search. When $\theta_{2}$ is given, we tackle the inner layer (13) to obtain the optimal transmit power allocation at SU and SJ. It is obvious that (13a) is monotonically increasing with respect to $P_{SJ}$. Meanwhile, considering the energy causality constraint in (13d) and transmit power constraint in (13e), the $P_{SJ}$ can be replaced by $P_{SJ}^{*}$, which is given by

(14) $$P_{SJ}^{*}=\min\left({\left[\frac{\eta \left(1-\theta_{2}\right)P_{S}h_{pj}}{\theta_{2}}\right]}^{+},\;I\right).$$

For the convenience of description, we utilize Logarithmic conversion formula $\log_{2}x=\frac{\ln x}{\ln2}$ to re-write (13) as

(15) $$\begin{array}{cc} (P3):\; & \max_{P_{SU}}\;\;\ln\left(P_{SU}h_{sr}+\sigma_{S}^{2}\right)-\ln\sigma_{S}^{2}-\ln\left(P_{SU}h_{se}+P_{SJ}^{*}h_{je}+\sigma_{E}^{2}\right),\\ \; & s.t.\;(13b),\;(13c). \end{array}$$

Specially, $-\ln\sigma_{S}^{2}$ can be removed from (15) as it does not affect the analysis results of the original problem. Then, (15) is re-expressed as $\ln\left(P_{SU}h_{sr}+\sigma_{S}^{2}\right)-\ln\left(P_{SU}h_{se}+P_{SJ}^{*}h_{je}+\sigma_{E}^{2}\right)$. It is observed that both $\ln\left(P_{SU}h_{sr}+\sigma_{S}^{2}\right)$ and $\ln\left(P_{SU}h_{se}+P_{SJ}^{*}h_{je}+\sigma_{E}^{2}\right)$ are concave. Thus, (15) can be represented as concave function minus concave function, which obviously corresponds to the difference of concave (DC) function. Theoretically, the optimal solution of such objective function can be obtained by DC programming, which is to successively solve the first-order Taylor approximation of the original problem.

### 3.1. DC Programming for (P3)

Based on DC theory, in order to convert the objective function of problem (P3) to an approximate convex problem, first-order Taylor approximation is used to approximate concave function $\ln\left(P_{SU}h_{se}+P_{SJ}^{*}h_{je}+\sigma_{E}^{2}\right)$ into linear form. Then, at the (*k* + 1)-th iteration of DC programming, let $\ln\left(P_{SU}h_{se}+P_{SJ}^{*}h_{je}+\sigma_{E}^{2}\right)$ be substituted by its first-order Taylor approximation expression as shown below

(16) $$\begin{array}{cc} \; & \ln\left(P_{SU}h_{se}+P_{SJ}^{*}h_{je}+\sigma_{E}^{2}\right)\\ \approx & \frac{h_{se}\left(P_{SU}-P_{SU}^{\left(k\right)}\right)}{P_{SU}^{\left(k\right)}h_{se}+P_{SJ}^{*}h_{je}+\sigma_{E}^{2}}\\ \; & +\ln\left(P_{SU}^{\left(k\right)}h_{se}+P_{SJ}^{*}h_{je}+\sigma_{E}^{2}\right),\; \end{array}$$

where $P_{SU}^{\left(k\right)}$ denotes the optimal transmit power at SU at the *k*-th iteration of DC programming, which will be used for the (k+1)-th iteration.

The objective function of (P3) is re-rewritten by replacing $\ln\left(P_{SU}h_{se}\;\;+\;\;P_{SJ}^{*}h_{je}\;\;+\;\;\sigma_{E}^{2}\right)$ with its first-order Taylor approximation expression represented at (16) and removing the constant terms. Then, we have

(17) $$\begin{array}{cc} (P4):\;\;\max_{P_{SU}} & \ln\left(P_{SU}h_{sr}+\sigma_{S}^{2}\right)+\ln\left(P_{SJ}^{*}h_{je}+\sigma_{E}^{2}\right)\\ \; & -\frac{h_{se}P_{SU}}{P_{SJ}^{*}h_{je}+P_{SU}^{\left(k\right)}h_{se}+\sigma_{E}^{2}}.\\ s.t.\;\; & (13b),\;(13c). \end{array}$$

In the objective function (17) of problem (P4), the first term is concave, the second term is constant, and the third term is linear with respect to $P_{SU}$, thus the objective function of problem (P4) is concave. Moreover, the constraints (13b) is affine, and (13c) is explicit constraint. Therefore, problem (P4) is a convex optimization problem. Based on DC programming, as the number of iteration increases, the objective function (17) of problem (P4) can successively converge to the original function.

### 3.2. The Lagrange Dual Method for (P4)

For each iteration in DC programming, we use Lagrange dual method to solve problem (P4). For convenience, we first make one substitution. Let

(18) $$a=\frac{h_{se}}{P_{SJ}^{*}h_{je}+P_{SU}^{\left(k\right)}h_{se}+\sigma_{E}^{2}}.$$

The partial Lagrangian of (17) is given by

(19) $$\begin{array}{cc} L(P_{SU},\;\mu )= & \left[\ln\left(P_{SU}h_{sr}+\sigma_{S}^{2}\right)+\ln\left(P_{SJ}^{*}h_{je}+\sigma_{E}^{2}\right)-aP_{SU}\right]\\ \; & +\mu (\eta \left(1-\theta_{2}\right)P_{SU}h_{ps}-\theta_{2}P_{SU}), \end{array}$$

where μ≥0 is the dual variable associated with the constraint (13b). The dual function is given by

(20) $$\begin{array}{cc} g\left(\mu \right)=\max_{P_{SU}}\;\; & L(P_{SU},\;\mu ),\\ s.t.\;\;\; & \left(13c\right). \end{array}$$

When μ is given, we can obtain the optimal solution of $P_{SU}$ as follows. The derivative of (19) with respect to $P_{SU}$ is expressed as

(21) $$\begin{array}{cc} \frac{\partial L}{\partial P_{SU}}= & \frac{h_{sr}}{P_{SU}h_{sr}+\sigma_{S}^{2}}-a-\mu \theta_{2}, \end{array}$$

and by setting (21) to zero, we have

(22) $$P_{SU}^{{}^{\prime }}=\frac{1}{a+\mu \theta_{2}}-\frac{\sigma_{S}^{2}}{h_{sr}}.$$

Considering the transmit power constraint in (13c), the optimal solution of $P_{SU}$ is expressed as

(23) $$\begin{array}{c} P_{SU}^{*}=\min\left({\left[P_{SU}^{{}^{\prime }}\right]}^{+},\;I\right). \end{array}$$

In order to minimize g(μ), we use the sub-gradient method to update the Lagrange multiplier, which is given by

(24) $$\begin{array}{c} \mu^{l+1}={\left[\mu^{l}\;\;-\;\;\Theta \left(\left(1-\theta_{2}\right)\eta P_{S}h_{ps}-\theta_{2}P_{SU}^{*}\right)\right]}^{+}, \end{array}$$

where Θ is the step-size, $P_{SU}^{*}$ is the optimal power allocation at the *l*-th iteration. Thus, we can obtain the optimal Lagrange multiplier and the optimal transmit power allocation at SU and SJ, which are denoted by $\mu^{*}$, $P_{SU}^{*}$ and $P_{SJ}^{*}$, respectively. Above all, Algorithm 1 summarizes the process of finding the optimal solution of problem (P1).

**Algorithm 1:** Optimal time allocation and transmit power allocation for (P1)

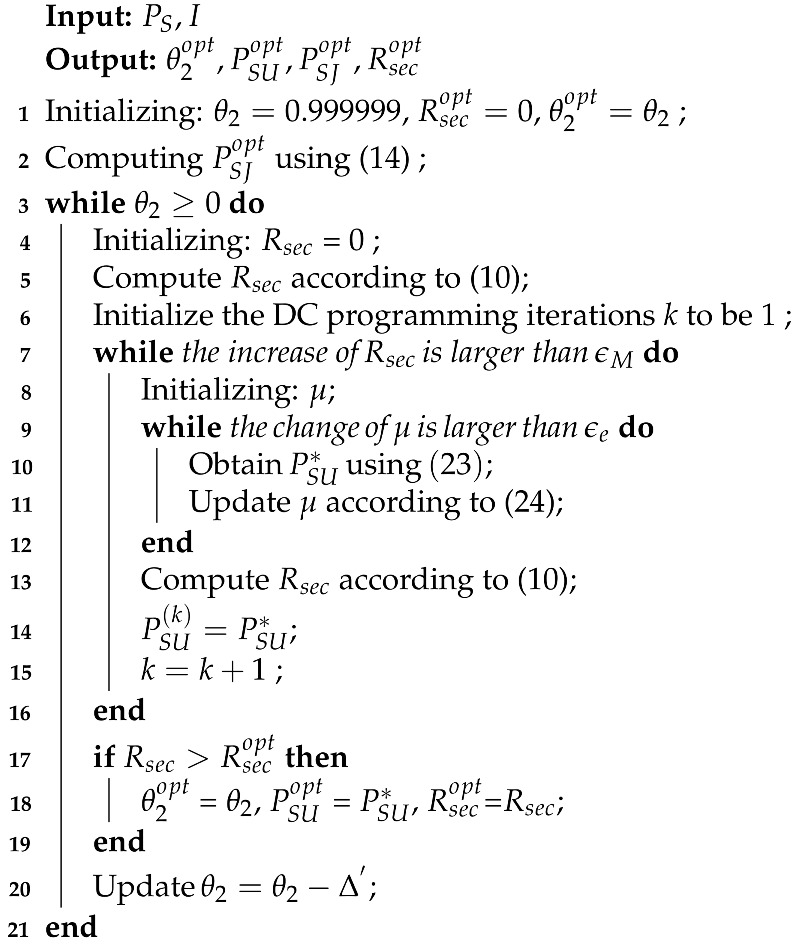



### 3.3. Computation Complexity

The computation complexity of Algorithm 1 is $O(N_{Iter}*N^{3})$, which is explained below. In the outer layer, the computation complexity of one-dimensional search can be ignored, because it is constant. Denote the number of iteration for the DC programming by $N_{Iter}$. At each iteration, the sub-gradient method is used to obtain the optimal transmit power allocation for problem (P4), which has the complexity $O(N^{3})$.

## 4. System Model and Problem Formulation for Underlay CR Networks with Multiple PUs

### 4.1. System Model

In this section, we consider a more general scenario with multiple PUs corresponding to their respective PR. In the secondary network, SU simultaneously shares spectrum with multiple PUs without causing harmful interference to them, where the confidential information is overheard by a potential eavesdropper. Similar to the scenario with one PU, a SJ is introduced to secure the secondary network. In the ET phase, SU and SJ harvest energy from the PUs transmissions. The energy captured by the SU is expressed as

(25) $$E_{SU}=\theta_{1}\eta \sum_{n=1}^{N}P_{S,n}h_{ps,n},\;n=1,\;2,\;\ldots ,\;N,$$

where $P_{S,n}$ and $h_{ps,n}$ denote the transmit power at the *n*-th PU and the channel power gain from the *n*-th PU to the SU, respectively. Similarly, the energy harvested by SJ is expressed as

(26) $$E_{SJ}=\theta_{1}\eta \sum_{n=1}^{N}P_{S,n}h_{pj,n},\;n=1,\;2,\;\ldots ,\;N,$$

where $h_{pj,n}$ is the channel power gain from the *n*-th PU to the SJ.

In the IT phase, the SU uses the harvested energy $E_{SU}$ to transmit confidential information to SR. The energy causality constraint at the SU should satisfy

(27) $$\theta_{2}\sum_{n=1}^{N}P_{SU,n}\leq \theta_{1}\eta \sum_{n=1}^{N}P_{S,n}h_{ps,n},$$

where $P_{SU,n}$ denotes the transmit power at the SU on the *n*-th PU spectrum. To guarantee the QoS requirement of the PUs, the transmit power $P_{SU,n}$ at the SU should be below a given threshold $I_{n}$, which is given by

(28) $$P_{SU,n}\leq I_{n}.$$

Meanwhile, the SJ simultaneously utilizes the harvested energy $E_{SJ}$ to generate the jamming signals to the eavesdropper. The energy causality and transmit power constraints at the SJ should satisfy

(29) $$\theta_{2}\sum_{n=1}^{N}P_{SJ,n}\leq \theta_{1}\eta \sum_{n=1}^{N}P_{S,n}h_{pj,n}$$

and

(30) $$P_{SJ,n}\leq I_{n},$$

where $P_{SJ,n}$ denotes the tansmit power at the SJ on the *n*-th PU spectrum.

Based on the above analysis, the throughput $R_{SR,n}$ at SR and the throughput $R_{SE,n}$ at the eavesdropper on the *n*-th PU spectrum, are expressed as

(31) $$R_{SR,n}=\theta_{2}W\log_{2}\left(1+\frac{P_{SU,n}h_{sr,n}}{\sigma_{S}^{2}}\right)$$

and

(32) $$R_{SE,n}=\theta_{2}W\log_{2}\left(1+\frac{P_{SU,n}h_{se,n}}{P_{SJ,n}h_{je,n}+\sigma_{E}^{2}}\right),$$

where $h_{sr,n}$, $h_{se,n}$ and $h_{je,n}$ denote the channel power gain from SU to SR, SU to the eavesdropper, and the jammer to the eavesdropper on the *n*-th PU spectrum, respectively. Therefore, the secrecy rate of the secondary network is given by

(33) $$R_{sec}=\sum_{n=1}^{N}{\left[R_{SR,n}-R_{SE,n}\right]}^{+}.$$

### 4.2. Problem Formulation

We target to maximize the secrecy rate in (33), subject to the time constraint in (1), the transmit power constraints in (28) and (30), and the energy causality constraints in (27) and (29). The secrecy rate maximization problem is formulated as

(34) (P5):maxθ1,θ2PSU,n,PSJ,n∑n=1Nθ2W[log2(1+PSU,nhsr,nσS2)−log2(1+PSU,nhse,nPSJ,nhje,n+σE2)],s.t. (1), (27), (28), (29), (30).

## 5. Secrecy Rate Optimization Design for Underlay CR Networks with Multiple PUs

Similar to the scenario with one PU, we translate problem (P5) into a nested form as

(35) $$\begin{array}{c} (P6):\;\max_{\theta_{2}}\;\;\theta_{2}WR\left(\theta_{2}\right),\\ s.t.\;\;0\leq \theta_{2}<1, \end{array}$$

where $R(\theta_{2})$ is expressed as

(36a) $$\begin{array}{cc} R\left(\theta_{2}\right)=\max_{P_{SU,n},P_{SJ,n}}\; & \sum_{n=1}^{N}\left[\log_{2}\left(1+\frac{P_{SU,n}h_{sr,n}}{\sigma_{S}^{2}}\right)-\log_{2}\left(1+\frac{P_{SU,n}h_{se,n}}{P_{SJ,n}h_{je,n}+\sigma_{E}^{2}}\right)\right], \end{array}$$

(36b) $$\begin{array}{cc} s.t.\;\;\;\;\;\;\;\; & \theta_{2}\sum_{n=1}^{N}P_{SU,n}\leq \left(1-\theta_{2}\right)\eta \sum_{n=1}^{N}P_{S,n}h_{ps,n}, \end{array}$$

(36c) $$\begin{array}{cc} \; & P_{SU,n}\leq I_{n}, \end{array}$$

(36d) $$\begin{array}{cc} \; & \theta_{2}\sum_{n=1}^{N}P_{SJ,n}\leq \left(1-\theta_{2}\right)\eta \sum_{n=1}^{N}P_{S,n}h_{pj,n}, \end{array}$$

(36e) $$\begin{array}{cc} \; & P_{SJ,n}\leq I_{n}. \end{array}$$

Here, for the outer layer (35), the optimal time allocation $\theta_{2}$ can be obtained through one-dimensional search. When $\theta_{2}$ is given, we solve the inner layer (36) to obtain the optimal transmit power allocation at SU and SJ on the *n*-th PU spectrum, respectively.

For the convenience of description, we utilize Logarithmic conversion formula to re-write (36) as

(37) $$\begin{array}{cc} (P7):\;\max_{P_{SU,n},\;P_{SJ,n}}\;\; & \sum_{n=1}^{N}[\ln\left(P_{SU,n}h_{sr,n}+\sigma_{S}^{2}\right)-\ln\sigma_{S}^{2}\\ \; & -\ln\left(P_{SU,n}h_{se,n}+P_{SJ,n}h_{je,n}+\sigma_{E}^{2}\right)+\ln\left(P_{SJ,n}h_{je,n}+\sigma_{E}^{2}\right)],\\ & s.t.\;(36b),\;(36c),\;(36d),\;(36e). \end{array}$$

After removing the constant term $-\ln\sigma_{S}^{2}$, (37) is re-expressed as $\sum_{n=1}^{N}[\ln\left(P_{SU,n}h_{sr,n}+\sigma_{S}^{2}\right)$

$-\ln\left(P_{SU,n}h_{se,n}+P_{SJ,n}h_{je,n}+\sigma_{E}^{2}\right)+\ln\left(P_{SJ,n}h_{je,n}+\sigma_{E}^{2}\right)]$. It is observed that both $\ln(P_{SU,n}h_{sr,n}$

$+\sigma_{S}^{2})+\ln\left(P_{SJ,n}h_{je,n}+\sigma_{E}^{2}\right)$ and $\ln\left(P_{SU,n}h_{se,n}+P_{SJ,n}h_{je,n}+\sigma_{E}^{2}\right)$ are concave. Thus, (37) can be represented as concave function minus concave function, which obviously corresponds to the difference of concave (DC) function.

### 5.1. DC Programming for (P7)

In order to convert the objective function of problem (P7) to an approximate convex problem, first-order Taylor approximation is used to approximate concave function $\ln\left(P_{SU,n}h_{se,n}+P_{SJ,n}h_{je,n}+\sigma_{E}^{2}\right)$ into linear form. Then, at the (*k* + 1)-th iteration of DC programming, let $\ln\left(P_{SU,n}h_{se,n}+P_{SJ,n}h_{je,n}+\sigma_{E}^{2}\right)$ be replaced by its first-order Taylor approximation expression as shown below

(38) $$\begin{array}{cc} \; & \ln\left(P_{SU,n}h_{se,n}+P_{SJ,n}h_{je,n}+\sigma_{E}^{2}\right)\\ \approx & \frac{h_{se,n}\left(P_{SU,n}-P_{SU,n}^{\left(k\right)}\right)+h_{je,n}\left(P_{SJ,n}-P_{SJ,n}^{\left(k\right)}\right)}{P_{SU,n}^{\left(k\right)}h_{se,n}+P_{SJ,n}^{\left(k\right)}h_{je,n}+\sigma_{E}^{2}}\\ \; & +\ln\left(P_{SU,n}^{\left(k\right)}h_{se,n}+P_{SJ,n}^{\left(k\right)}h_{je,n}+\sigma_{E}^{2}\right),\; \end{array}$$

where $P_{SU,n}^{\left(k\right)}$ and $P_{SJ,n}^{\left(k\right)}$ denote the optimal transmit power at SU and SJ on the *n*-th PU spectrum at the *k*-th iteration of DC programming, which will be used for the (k+1)th iteration.

The objective function of (P7) is re-written by replacing $\ln\left(P_{SU,n}h_{se,n}+P_{SJ,n}h_{je,n}+\sigma_{E}^{2}\right)$ with its first-order Taylor approximation expression represented at (38) and removing the constant term. Then, we have

(39) $$\begin{array}{cc} (P8):\;\;\max_{P_{SU,n},\;P_{SJ,n}} & \sum_{n=1}^{N}[\ln\left(P_{SU,n}h_{sr,n}+\sigma_{S}^{2}\right)+\ln\left(P_{SJ,n}h_{je,n}+\sigma_{E}^{2}\right)\\ \; & -\frac{P_{SU,n}h_{se,n}+P_{SJ,n}h_{je,n}}{P_{SJ,n}^{\left(k\right)}h_{je,n}+P_{SU,n}^{\left(k\right)}h_{se,n}+\sigma_{E}^{2}}].\\ s.t.\;\; & (36b),\;(36c),\;(36d),\;(36e). \end{array}$$

Similar to the objective function (17) of problem (P4), the objective function (39) of problem (P8) is a convex optimization problem. Based on DC programming, the objective function (39) of problem (P8) can successively converge to the original problem as the number of iterations increases.

### 5.2. The Lagrange Dual Method for (P8)

For each iteration in DC programming, we use Lagrange dual method to solve problem (P8). For convenience, we first make some substitutions. Let

(40) $$a_{n}=\frac{h_{je,n}}{P_{SJ,n}^{\left(k\right)}h_{je,n}+P_{SU,n}^{\left(k\right)}h_{se,n}+\sigma_{E}^{2}}$$

and

(41) $$b_{n}=\frac{h_{se,n}}{P_{SJ,n}^{\left(k\right)}h_{je,n}+P_{SU,n}^{\left(k\right)}h_{se,n}+\sigma_{E}^{2}}.$$

The partial Lagrangian of (39) is given by

(42) $$\begin{array}{cc} L(P_{SU,n},\;P_{SJ,n},\;\lambda ,\;\mu )= & \sum_{n=1}^{N}\left[\ln\left(P_{SU,n}h_{sr,n}+\sigma_{S}^{2}\right)+\ln\left(P_{SJ,n}h_{je,n}+\sigma_{E}^{2}\right)-a_{n}P_{SJ,n}-b_{n}P_{SU,n}\right]\\ \; & +\lambda (\eta \left(1-\theta_{2}\right)\sum_{n=1}^{N}P_{S,n}h_{pj,n}-\theta_{2}\sum_{n=1}^{N}P_{SJ,n})\\ \; & +\mu (\eta \left(1-\theta_{2}\right)\sum_{n=1}^{N}P_{S,n}h_{ps,n}-\theta_{2}\sum_{n=1}^{N}P_{SU,n}), \end{array}$$

where λ≥0 and μ≥0 are the dual variables associated with the constraints (36d) and (36b). The dual function is given by

(43) $$\begin{array}{cc} g\left(\lambda ,\;\mu \right)=\max_{P_{SU,n},\;P_{SJ,n}}\;\; & L(P_{SU,n},\;P_{SJ,n},\;\lambda ,\;\mu ),\\ s.t.\;\;\; & \left(36c\right),\;\left(36e\right). \end{array}$$

When λ and μ are given, we can obtain the solutions of $P_{SU,n}$ and $P_{SJ,n}$ as follows. The derivative of (42) with respect to $P_{SU,n}$ is expressed as

(44) $$\begin{array}{cc} \frac{\partial L}{\partial P_{SU,n}}= & \frac{h_{sr,n}}{P_{SU,n}h_{sr,n}+\sigma_{S}^{2}}-b_{n}-\mu \theta_{2}, \end{array}$$

and by setting (44) to zero, we have

(45) $$P_{SU,n}^{{}^{\prime }}=\frac{1}{b_{n}+\mu \theta_{2}}-\frac{\sigma_{S}^{2}}{h_{sr,n}}.$$

Similarly, the derivative of (42) with respect to $P_{SJ,n}$ is expressed as

(46) $$\begin{array}{cc} \frac{\partial L}{\partial P_{SJ,n}}= & \frac{h_{je,n}}{P_{SJ,n}h_{je,n}+\sigma_{E}^{2}}-a_{n}-\lambda \theta_{2}, \end{array}$$

and by setting (46) to zero, we have

(47) $$P_{SJ,n}^{{}^{\prime }}=\frac{1}{a_{n}+\lambda \theta_{2}}-\frac{\sigma_{E}^{2}}{h_{je,n}}.$$

Considering the transmit power constraints in (36c) and (36e), the optimal solutions of $P_{SU,n}$ and $P_{SJ,n}$ are expressed as

(48) $$\begin{array}{c} P_{SU,n}^{*}=\min\left({\left[P_{SU,n}^{{}^{\prime }}\right]}^{+},\;I_{n}\right), \end{array}$$

and

(49) $$\begin{array}{c} P_{SJ,n}^{*}=\min\left({\left[P_{SJ,n}^{{}^{\prime }}\right]}^{+},\;I_{n}\right). \end{array}$$

To minimize g(λ,μ), we use the sub-gradient method to update Lagrange multipliers λ and μ, which are given by

(50) $$\begin{array}{c} \lambda^{l+1}={\left[\lambda^{l}\;\;-\;\;\Theta \left(\left(1-\theta_{2}\right)\eta \sum_{n=1}^{N}P_{S,n}h_{pj,n}-\theta_{2}\sum_{n=1}^{N}P_{SJ,n}^{*}\right)\right]}^{+} \end{array}$$

and

(51) $$\begin{array}{c} \mu^{l+1}={\left[\mu^{l}\;\;-\;\;\Theta \left(\left(1-\theta_{2}\right)\eta \sum_{n=1}^{N}P_{S,n}h_{ps,n}-\theta_{2}\sum_{n=1}^{N}P_{SU,n}^{*}\right)\right]}^{+}, \end{array}$$

where Θ is the step-size, $P_{SU,n}^{*}$ and $P_{SJ,n}^{*}$ are the optimal power allocation solutions at the *l*-th iteration. Thus, we can obtain the optimal Lagrange multipliers and the optimal transmit power allocation at SU and SJ, which are denoted by $\lambda^{*}$, $\mu^{*}$, $P_{SU}^{*}$, and $P_{SJ}^{*}$, respectively. The algorithm for problem (P8) can be referred to Algorithm 1. For reasons of space, we do not elaborate on it here.

## 6. Numerical Results and Discussions

In this section, we demonstrate the performance of the proposed schemes by comparing them with the following two benchmark schemes.

- There is no cooperative jammer in the CR networks. The time allocation of ET phase and IT phase, and the transmit power allocation at the SU are optimized. This scheme is denoted as “noCJ”.
- Time allocation is evenly divided between ET phase and IT phase. That is, only the transmit power at SU and SJ are optimized. This scheme is denoted as $\theta_{2}=0.5$.
- Time allocation is evenly divided between ET phase and IT phase. For the scenario with one PU, SU uses up all the harvested energy. This scheme is denoted as $\theta_{2}=0.5$, $P_{SU}=P_{SU}^{MAX}$, where $P_{SU}^{MAX}=\min\left\{\frac{E_{SU}}{\theta_{2}},I\right\}$. For the scenario with multiple PUs, the SU and SJ use up all the harvested energy, and the transmission power of the SU and SJ on each sub channel is equally distributed, respectively. This scheme is denoted as $\theta_{2}=0.5,P_{SU,n}=\frac{P_{SU}^{MAX}}{N},P_{SJ,n}=\frac{P_{Sj}^{MAX}}{N}$, where $\frac{P_{SU}^{MAX}}{N}=\min\left\{\frac{E_{SU}}{\theta_{2}N},I_{n}\right\}$ and $\frac{P_{SJ}^{MAX}}{N}=\min\left\{\frac{E_{SJ}}{\theta_{2}N},I_{n}\right\}$.

In the simulations, the bandwidth is set to be 10MHZ. The interference power threshold of the PU is $0.5\times 10^{-4}$w. The noise power at SR and the eavesdropper are set to be the same, where $\sigma_{E}^{2}=\sigma_{S}^{2}=10^{-12}$w. The channel power gain is modeled as $10^{-3}d^{-3}$, where *d* is the distance between the transmitter and the receiver. For the sub-gradient method, the initial dual variables λ and μ are both set to be 100. For the CR networks with multiple PUs, the number of the PUs is 2, and the transmit power at each PU is uniform.

### 6.1. The Secrecy Rate Evaluation for CR Networks with One PU

**Figure 3:**
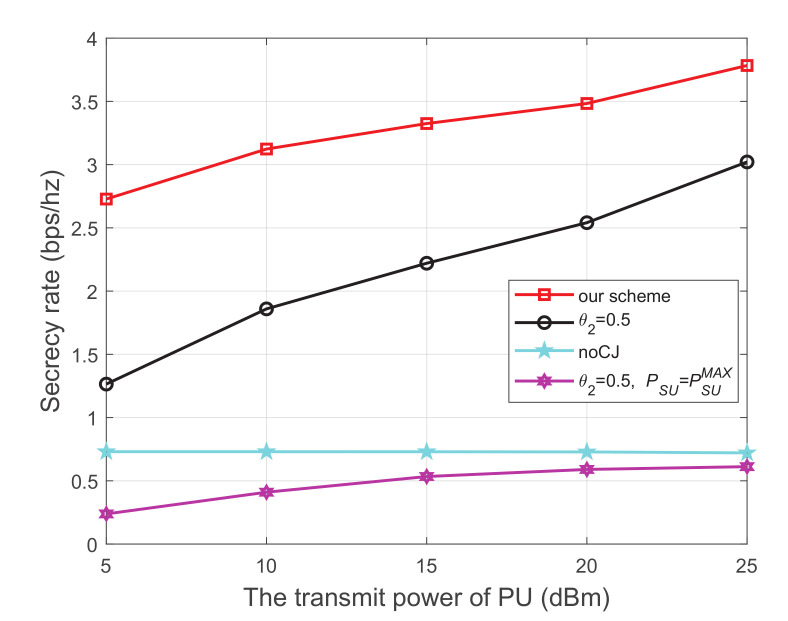
The secrecy rate versus transmit power of PU.

Figure 3 shows the secrecy rate versus the transmit power of the PU, where the energy harvest efficiency η is 0.5, and the distance from PU to SU, PU to SJ, SU to SR, SU to the eavesdropper, and SJ to the eavesdropper are 5 m, 4 m, 3 m, 4 m, and 3 m, respectively. We have the following observations from Figure 3. First, the secrecy performance of our scheme is obviously superior to the other three schemes. Second, for each scheme using the cooperative jammer, the secrecy rate monotonically increases with the increase of the transmit power of the PU. This is because both SU and SJ harvest energy from the primary network. When the transmit power at the PU increases, more energy will be captured to transmit information and generate interference, which simultaneously improves the channel quality of the main link and impairs that of the wiretap link, respectively. Thirdly, when the cooperative jammer is not introduced, the secrecy rate is relatively low and the growth trend is not obvious. We can infer from the trend of the noCJ scheme that setting the SU to a larger transmit power does not necessarily result in a greater secrecy rate. This is because the larger transmit power at SU will simultaneously improve the channel quality of the main link and the wiretap link.

**Figure 4:**
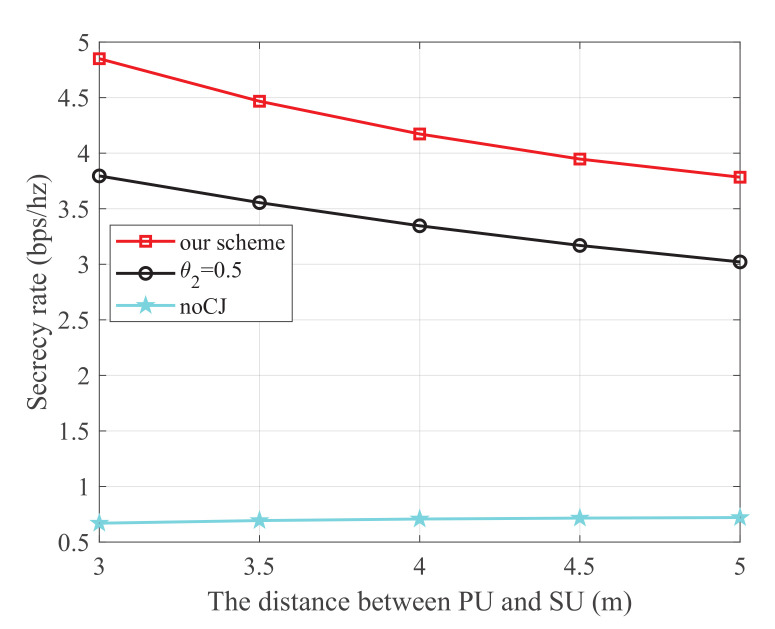
The secrecy rate versus the distance between PU and SU.

Figure 4 shows the secrecy rate versus the distance between PU and SU, where the energy harvest efficiency η is 0.5, the transmit power at PU is 25 dBm, and the distance from PU to SJ, SU to SR, SU to the eavesdropper, and SJ to the eavesdropper are 4 m, 3 m, 4 m, and 3 m, respectively. It is observed that our scheme is superior to the other two schemes. Additionally, for the scheme using the cooperative jammer, the secrecy rate monotonically decreases with the increase of the distance between PU and SU. This is because SU harvests energy from the PU transmissions. When the distance between PU and SU increases, less energy will be harvested to transmit information, which degrades the main link.

**Figure 5:**
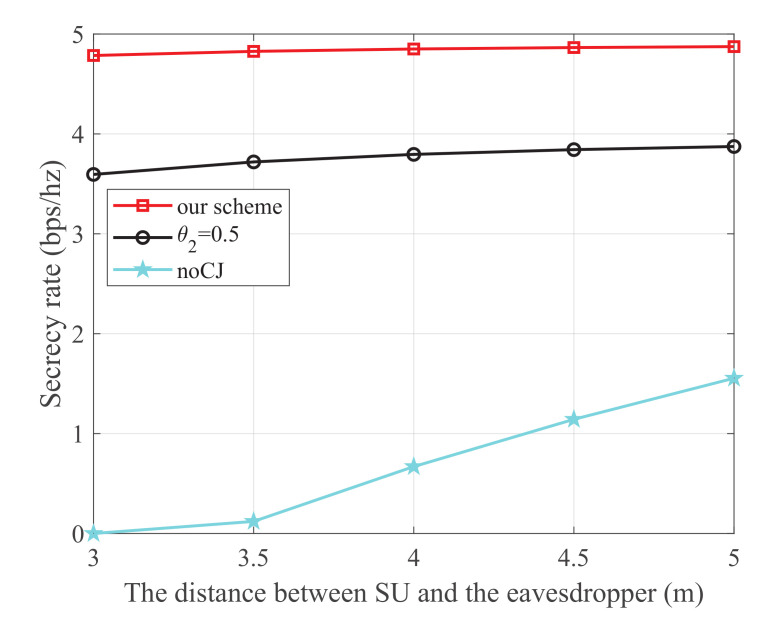
The secrecy rate versus the distance between SU and the eavesdropper.

Figure 5 shows the secrecy rate versus the distance between SU and the eavesdropper, where the energy harvest efficiency η is 0.5, the transmit power at PU is 25 dBm, and the distance from PU to SU, PU to SJ, SU to SR, and SJ to the eavesdropper are 3 m, 4 m, 3 m, and 3 m, respectively. It is observed that our scheme is superior to the other two schemes. Moreover, for the noCJ scheme, the secrecy rate monotonically increases with the increase of the distance between SU and the eavesdropper. This is because the wiretap link is degraded as the distance between SU and eavesdropper increases.

**Figure 6:**
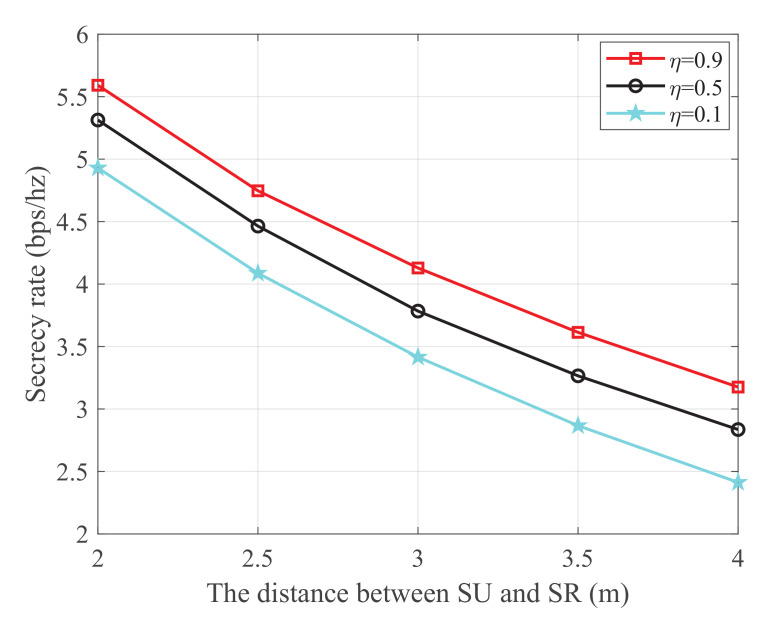
The secrecy rate versus the distance between SU and SR.

Figure 6 shows the secrecy rate versus the distance between SU and SR with different energy harvest efficiency, where the transmit power at PU is 25 dBm, the distance from PU to SU, PU to SJ, and SJ to the eavesdropper are 2 m, 4 m, and 3 m, respectively. It is observed that the secrecy rate monotonically decreases with the increase of the distance between SU and SR. This is because the main link is degraded as the distance between SU and SR increases. Additionally, it is also observed that the secrecy rate increases with the increase of the energy harvest efficiency. This is because both SU and SJ harvest energy from the PU transmissions. When the energy harvest efficiency η increases, more energy will be captured to transmit information and generate jamming signals, which simultaneously improves the channel quality of the main link and impairs that of the wiretap link, respectively.

### 6.2. The Secrecy Rate Evaluation for CR Networks with Multiple PUs

**Figure 7:**
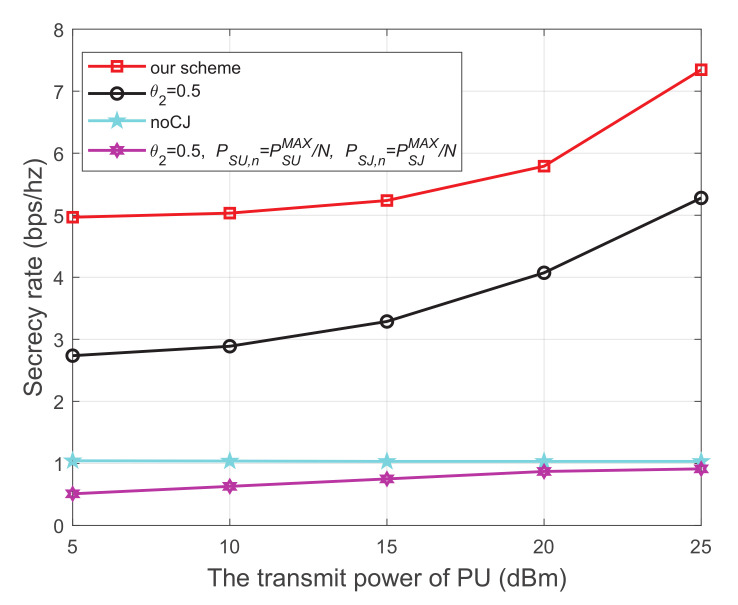
The secrecy rate versus the transmit power of PU.

Figure 7 shows the secrecy rate versus the transmit power of the PU, where the energy harvest efficiency η is 0.5, and the distance from the 1-th PU to SU, the 2-th PU to SU, the 1-th PU to SJ, the 2-th PU to SJ, SU to SR, SU to the eavesdropper, and SJ to the eavesdropper are 5 m, 3 m, 4 m, 5 m, 3 m, 3 m, and 3 m, respectively. Figure 7 shows that similar to the CR networks with one PU, the secrecy rate monotonously increases with the transmit power of PUs.

**Figure 8:**
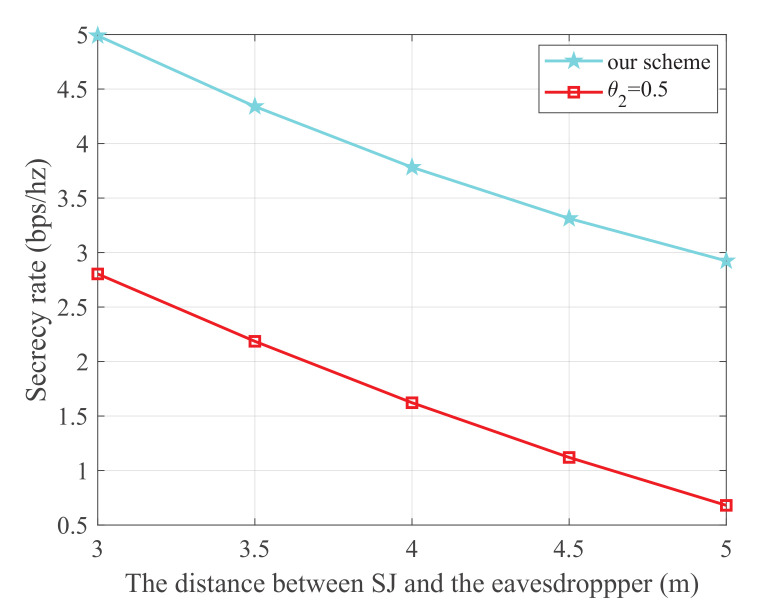
The secrecy rate versus the distance between SJ and the eavesdropper.

Figure 8 shows the secrecy rate versus the distance between SJ and the eavesdropper, where the energy harvest efficiency η is 0.5, the transmit power of the PU is 25 dBm, and the distance from the 1-th PU to SU, 2-th PU to SU, 1-th PU to SJ, 2-th PU to SJ, SU to SR, SU to the eavesdropper, and SJ to the eavesdropper are 5 m, 3 m, 4 m, 5 m, 3 m, and 3 m, respectively. It is obvious that our scheme is superior to the $\theta_{2}=0.5$ scheme. We can also observe that the secrecy rate monotonically decreases with the increase in distance between SJ and the eavesdropper. This is because a smaller distance between SJ and the eavesdropper will further degrade the wiretap link.

**Figure 9:**
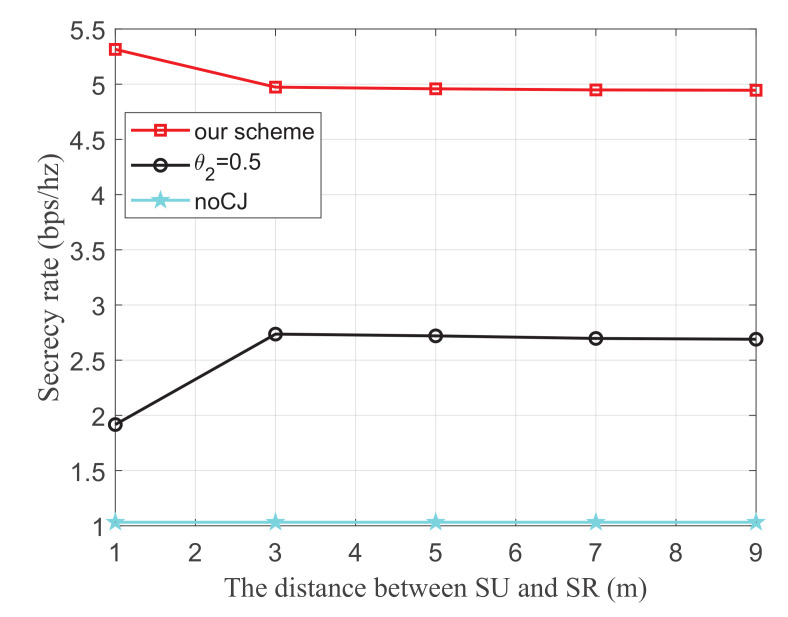
The secrecy rate versus the distance between SU and SR.

Figure 9 shows the secrecy rate versus the distance between SU and SR, where the energy harvest efficiency η is 0.5, the transmit power of the PU is 25 dBm, and the distance from the 1-th PU to SU, the 2-th PU to SU, the 1-th PU to SJ, the 2-th PU to SJ, SU to the eavesdropper, and SJ to the eavesdropper are 3 m, 3 m, 4 m, 5 m, 3 m, and 3 m, respectively. It is observed from our scheme that as the distance between SU and SR increases, the secrecy rate decreases obviously, and then it changes slowly. We can infer that when the distance between SU and SR increases to a certain extent, the distance of which does not cause large fluctuation on secrecy rate.

**Figure 10:**
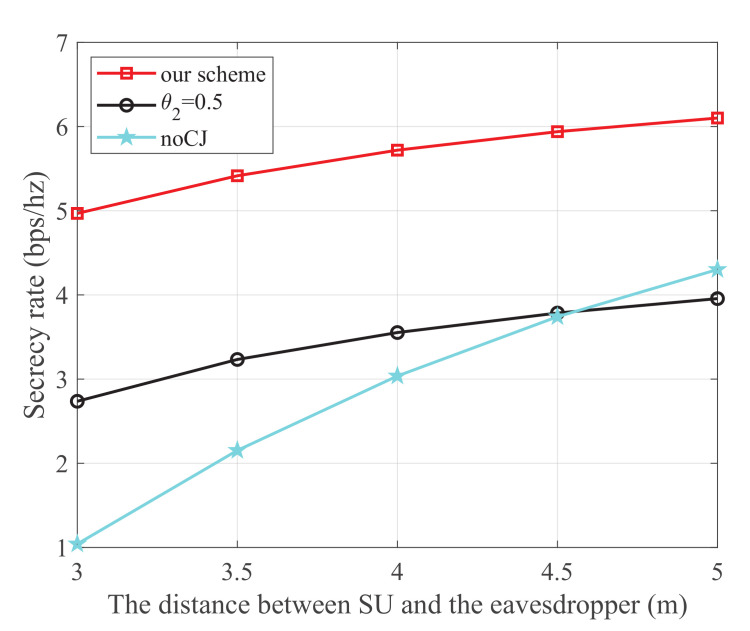
The secrecy rate versus the distance between SU and the eavesdropper.

Figure 10 shows the secrecy rate versus the distance between SU and the eavesdropper, where the energy harvest efficiency η is 0.5, the transmit power of each PU is 25 dBm, and the distance from the 1-th PU to SU, the 2t-h PU to SU, the 1-th PU to SJ, the 2-th PU to SJ, SU to SR, and SJ to the eavesdropper are 5 m, 3 m, 4 m, 5 m, 3 m, and 3 m, respectively. We can see that similar conclusions can be drawn as those of Figure 5.

## 7. Conclusions

For the underlay CR networks with one PU as well as multiple PUs, this paper studied the PLS of the secondary network while without causing the harmful interference to the primary networks. We introduce a wireless powered cooperative jammer to generate jamming signals to guarantee the information privacy for the secondary network. We first proposed communication schemes for this type of CR networks. Then, we investigated the secrecy rate maximization problems by jointly optimizing the time allocation between the ET phase and IT phase, and the transmit power allocation for SU and SJ. We designed efficient nested form algorithms which are applicable to both two non-convex problems. The numerical results verified that the proposed schemes significantly improve the secrecy performance of the secondary network, as compared to the benchmark schemes.
